# Estimating the burden of respiratory syncytial virus (RSV) on respiratory hospital admissions in children less than five years of age in England, 2007‐2012

**DOI:** 10.1111/irv.12443

**Published:** 2017-01-21

**Authors:** Rachel Melanie Reeves, Pia Hardelid, Ruth Gilbert, Fiona Warburton, Joanna Ellis, Richard G. Pebody

**Affiliations:** ^1^ Farr Institute of Health Informatics ResearchLondonUK; ^2^ Institute of Child HealthUniversity College LondonLondonUK; ^3^ Respiratory Diseases DepartmentPublic Health EnglandColindaleLondonUK; ^4^ Statistics and Modelling Economics DepartmentPublic Health EnglandColindaleLondonUK; ^5^ Virus Reference DepartmentPublic Health EnglandColindaleLondonUK

**Keywords:** bronchiolitis, bronchitis, child, England, hospital admissions, pneumonia, respiratory syncytial virus

## Abstract

**Background:**

Respiratory syncytial virus (RSV) is a leading cause of hospital admission in young children. With several RSV vaccines candidates undergoing clinical trials, recent estimates of RSV burden are required to provide a baseline for vaccine impact studies.

**Objectives:**

To estimate the number of RSV‐associated hospital admissions in children aged <5 years in England over a 5‐year period from 2007 using ecological time series modelling of national hospital administrative data.

**Patients/Methods:**

Multiple linear regression modelling of weekly time series of laboratory surveillance data and Hospital Episode Statistics (HES) data was used to estimate the number of hospital admissions due to major respiratory pathogens including RSV in children <5 years of age in England from mid‐2007 to mid‐2012, stratified by age group (<6 months, 6‐11 months, 1‐4 years) and primary diagnosis: bronchiolitis, pneumonia, unspecified lower respiratory tract infection (LRTI), bronchitis and upper respiratory tract infection (URTI).

**Results:**

On average, 33 561 (95% confidence interval 30 429‐38 489) RSV‐associated hospital admissions in children <5 years of age occurred annually from 2007 to 2012. Average annual admission rates were 35.1 (95% CI: 32.9‐38.9) per 1000 children aged <1 year and 5.31 (95% CI: 4.5‐6.6) per 1000 children aged 1‐4 years. About 84% (95% CI: 81‐91%) of RSV‐associated admissions were for LRTI. The diagnosis‐specific burden of RSV‐associated admissions differed significantly by age group.

**Conclusions:**

RSV remains a significant cause of hospital admissions in young children in England. Individual‐level analysis of RSV‐associated admissions is required to fully describe the burden by age and risk group and identify optimal prevention strategies.

## 1 Background

Respiratory syncytial virus (RSV) is a major cause of respiratory tract infections (RTI) worldwide.^1^ In older children and adults, RSV infection often leads to mild upper respiratory tract infection (URTI). However, in infants and young children, RSV is an important cause of severe respiratory infection, particularly bronchiolitis, which may require hospital admission.^2^ With a number of RSV vaccine candidates now in phase 2 and 3 clinical trials, it is essential to have accurate estimates of the hospital burden of RSV by age and risk group in order to determine the potential benefits of a future vaccine programme.^3^

Calculating the national burden of disease due to RSV is not straightforward. A reliable diagnosis of RSV infection relies on the detection of RSV in respiratory secretions, but only a minority of children hospitalised with an acute respiratory infection will undergo laboratory testing to identify the causal pathogen.^4^ The vast majority of respiratory infections are therefore recorded in hospital admission data under non‐specific diagnosis such as unspecified pneumonia or bronchiolitis. Hospital admission data alone can therefore not be used to accurately calculate the burden of RSV in secondary care.

Respiratory pathogens have varying temporal patterns which can be observed using laboratory surveillance data. Statistical models which utilise the seasonal variation in laboratory reports by pathogen can be constructed to attribute hospital admissions to different viruses.^4^, ^5^ This method of estimating the hospital burden of RSV has previously been used in the UK; however, the most recent study only considers data up to 2009.^4^, ^5^, ^6^ Surveillance of respiratory viruses including RSV has been strengthened in England since the 2009 influenza A (H1N1) pandemic, with more widespread use of laboratory confirmation for respiratory viruses with PCR methods, and a greater degree of reporting to national surveillance schemes.^7^ In addition, a recent study in the UK demonstrates that hospital admission due to bronchiolitis is increasing over time.^8^ These developments emphasise the need for more recent estimates of RSV‐associated hospital admissions as previous estimates may not reflect the current burden of disease.

The aim of this work was to estimate the number of hospital admissions attributable to RSV in children <5 years of age in England in the period from mid‐2007 to mid‐2012 using ecological time series modelling of national laboratory surveillance and hospital administrative data.

## 2 Methods

### 2.1 Data sources

#### 2.1.1 National laboratory reports

The Second Generation Surveillance System (SGSS)—formerly LabBase2—is a microbiology laboratory surveillance database at the Centre for Infectious Disease Surveillance and Control (CIDSC) at Public Health England (PHE), the national centre responsible for infectious disease surveillance, prevention and control. Positive test results for microorganisms recorded in SGSS are collected from local pathology systems transmitted from PHE, NHS and private microbiology laboratories in England.^9^ All clinically significant microorganisms should be reported, although no guidelines for the judgement of clinical significance are defined. Weekly laboratory reports of samples positive for RSV, influenza A, influenza B, rhinovirus, parainfluenza, human metapneumovirus (hMPV), adenovirus, *Streptococcus pneumoniae, Mycoplasma pneumoniae* and *Haemophilus influenza* in children <5 years of age (at the time of the sample) in England from calendar week 27 (mid‐July) in 2007 to calendar week 28 (mid‐July) in 2012 were extracted. If individuals had multiple positive samples for the same virus within a two‐week period, only one record was included in this analysis—this was to avoid multiple tests in the same individual within the same infection episode being counted as separate infections.

#### 2.1.2 Hospital Episode Statistics (HES)

The Hospital Episode Statistics (HES) admitted patient care database, held by the Health and Social Care Information Centre (HSCIC), contains routinely collected data on all admissions to all NHS hospitals in England. Records include clinical, geographical and administrative information including admission and discharge dates, on every patient. In this analysis, an admission refers to a single HES spell—from admission to discharge in one hospital.

Diagnoses are recorded in HES using International Classification of Diseases 10^th^ Revision (ICD‐10) codes, with up to 20 diagnosis codes allowed per HES episode. Each admission is allocated a primary diagnosis which is the main reason for the length of stay in hospital. All admissions with a primary diagnosis of ICD‐10 codes for bronchiolitis (ICD‐10 J21), pneumonia (J12‐18), unspecified lower respiratory tract infection (LRTI) (J22), bronchitis (J20) and upper respiratory tract infection (URTI) (J00‐06) in children <5 years of age (at admission) in England from calendar week 27 in 2007 to calendar week 26 in 2012 were included in the study. Calendar weeks were defined as blocks of 7 days beginning on 1st January each year, with week 52 allowed to have more than 7 days. Only the primary diagnosis was included to avoid double counting of admissions which may have two or more of these diagnoses. HES data were not available for 2013 onwards. The weekly number of hospital admissions was stratified into three groups: <6 months, 6‐11 months and 1‐4 years.

### 2.2 Statistical analysis

In this study, we use the observed temporal variation in weekly laboratory reports of potential causative pathogens to estimate the number of RTI hospital admissions that could be attributed to RSV, building on methods applied in previous modelling studies.^4^, ^10^, ^11^ Separate models were developed for each primary diagnosis, using the weekly number of hospital admissions in children <5 years of age in England for each respective diagnosis as the dependent variable. For each diagnosis, separate models were constructed by age group (<6 months, 6‐11 months, 1‐4 years).

Multiple linear regression models were used to estimate the number of hospital admissions due to RSV from HES data coded as acute bronchiolitis, pneumonia, unspecified LRTI, bronchitis and URTI. These models have been used in similar studies.^4^, ^10^, ^12^, ^13^ All models used the weekly number of laboratory‐confirmed episodes in children <5 years of age in England for the following pathogens as the independent variables: RSV, influenza A, influenza B, rhinovirus, parainfluenza, human metapneumovirus (hMPV) and adenovirus. The weekly number of laboratory‐confirmed episodes of *Streptococcus pneumoniae, Mycoplasma pneumoniae* and *Haemophilus influenza* in children <5 years of age in England was also included as independent variables in the models for pneumonia and unspecified LRTI. All variables were first included in the models, then those with negative coefficients removed (in order of decreasing significance) due to biological implausibility (pathogens cannot cause a negative number of hospital admissions), followed by those that did not contribute significantly to the model (*F*‐test *P*>.05). Interactions between all pathogens in the final model were investigated (*P*≤.01 was considered significant) due to the potential for co‐circulation of pathogens. Interactions between pathogens in the final model and an indicator variable taking the value 0 for the pre‐pandemic period (before week 20 2009) and 1 for the pandemic and post‐pandemic period were investigated to account for potential changes in testing practice following the 2009 influenza pandemic (*P*≤.01 was considered significant).

Final estimates of the number of hospital admissions attributable to each pathogen (including 95% confidence intervals (CIs)) were calculated by multiplying the coefficient from the final model by the total number of weekly laboratory‐confirmed episodes for each relevant pathogen. The total number of hospital admissions for all children <5 years old was calculated as the sum of the number of hospital admissions calculated in each age group for each pathogen.

Admission rates were calculated using ONS mid‐year population estimates for England by age group (<1 year, 1‐4 years).^14^, ^15^ The average of ONS mid‐year population estimates for 2007 and 2008 was used as the denominator for the 2007/2008 epidemiological year and the average of ONS mid‐year population estimates for 2008 and 2009 used for the 2008/2009 epidemiological year, etc.

## 3 Results

### 3.1 Seasonality of laboratory reports and hospital admissions

**Figure 1:**
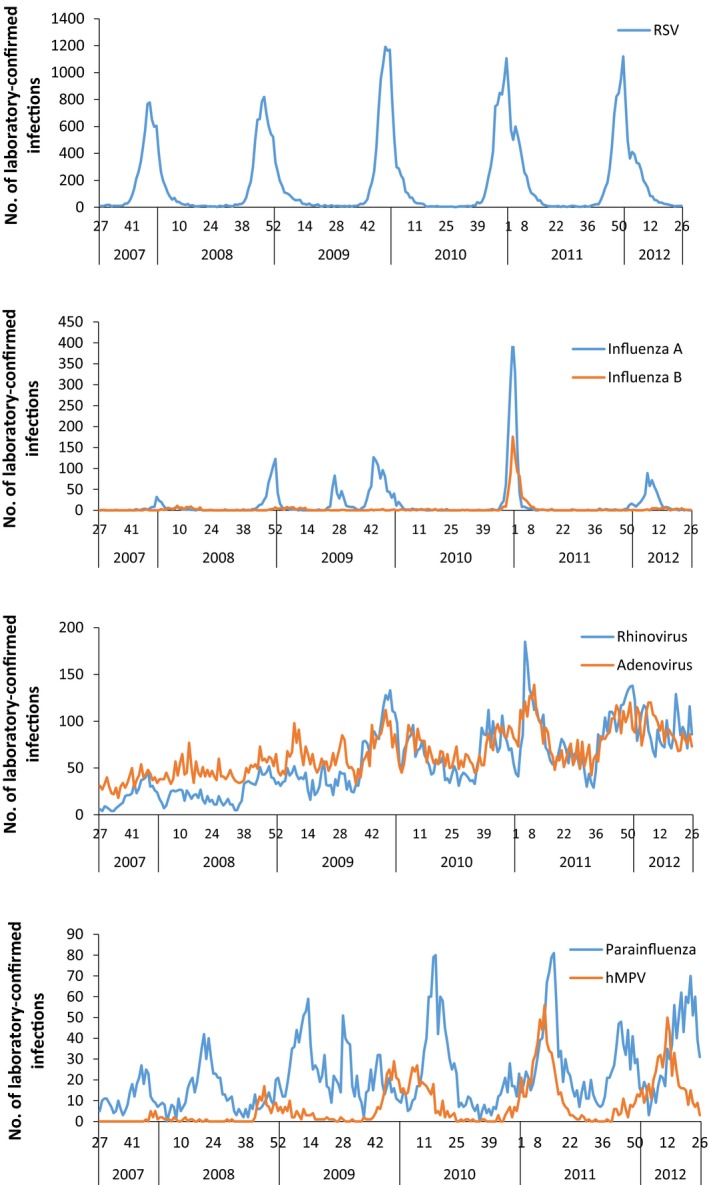
Weekly number of laboratory‐confirmed cases of major respiratory viruses recorded in SGSS for children <5 y of age, over time

**Figure 2:**
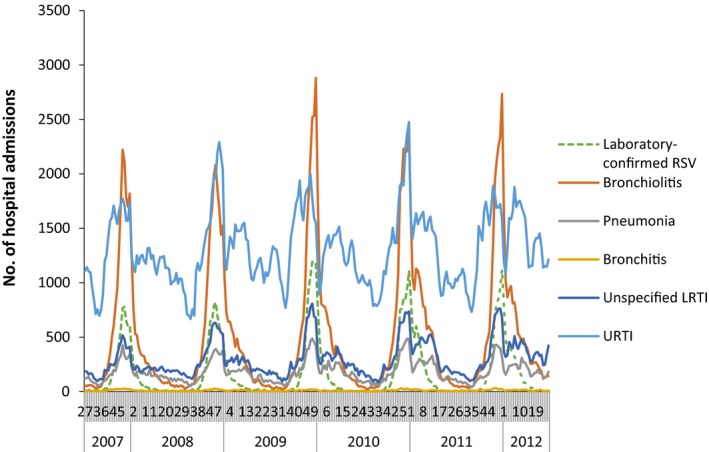
Weekly number of hospital admissions in children <5 y of age in England with any diagnosis of URTI, bronchiolitis, pneumonia, bronchitis or unspecified LRTI. Weekly number of laboratory‐confirmed RSV infections in children <5 y of age in England (SGSS) shown in orange

The temporal variation in laboratory reports by pathogen is shown in Figure 1. The temporal trends in hospital admissions for children <5 years old in England with a primary diagnosis of bronchiolitis, pneumonia, bronchitis, unspecified LRTI or URTI are shown in Figure 2. Hospital admissions with a primary diagnosis of bronchiolitis were markedly seasonal and mirror the pattern of laboratory‐confirmed RSV infections. Hospital admissions with a primary diagnosis of pneumonia had a very similar seasonal pattern to hospital admissions with a primary diagnosis of unspecified LRTI, with peaks also occurring at the same time as the peaks in laboratory‐confirmed RSV infections in SGSS each year (Figure 2).

### 3.2 Estimated RSV‐associated hospital admissions

#### 3.2.1 All RTI admissions

Of the 121 968 hospital admissions with a primary diagnosis of bronchiolitis, pneumonia, bronchitis, unspecified LRTI or URTI that occurred annually in children <5 years of age from mid‐2007 to mid‐2012, we estimate that 28% (33 561/121 968, 95% CI: 25‐32%) were due to RSV. Of RSV‐associated RTI hospital admissions, 48% (16 202/33 561, 95% CI: 46‐52%) were in children <6 months of age, 21% (7108/33 561, 95% CI: 19‐25%) were in children 6‐11 months of age, and 31% (10 251/33 561, 95% CI: 26‐38%) were in children aged 1‐4 years (Table 1). The majority, 84% (28 111/33 561, 95% CI: 81‐91%), of RSV‐associated RTI hospital admissions were for LRTI. About 65% (21 418/33 561, 95% CI: 62‐70%) of RSV‐associated hospital admissions were coded as bronchiolitis (Tables 1 and 2).

**Table 1:** Average annual number of hospital admissions in children <5 y of age in England: total per primary diagnosis and number estimated to be attributed to RSV by the final models, stratified by age group (<6 mo, 6‐11 mo, 1‐4 y)

Primary diagnosis	Annual number of hospital admissions (95% CI)			
	<6 mo	6‐11 mo	1‐4 y	Total
Bronchiolitis				
Total	18 246	7652	2071	27 969
RSV‐associated	14 962 (14 396‐15 942)	5319 (5066‐5775)	1549 (1467‐1631)	21 830 (20 929‐23 348)
Pneumonia				
Total	739	1294	7503	9537
RSV‐associated	138 (101‐158)	285 (245‐324)	1923 (1 718‐2 128)	2346 (2064‐2610)
Bronchitis				
Total	159	113	276	547
RSV‐associated	89 (76‐113)	41 (29‐62)	39 (23‐55)	169 (128‐230)
Unspecified LRTI				
Total	1224	2546	11 690	15 461
RSV‐associated	71 (37‐104)	429 (322‐619)	3266 (3007‐3525)	3766 (3366‐4248)
URTI				
Total	8868	12 737	46 850	68 455
RSV‐associated	942 (802‐1082)	1034 (742‐1559)	3474 (2398‐5412)	5450 (3222‐8053)
Total	29 236	24 342	68 390	121 968
Total RSV‐associated	16 202 (15 412‐17 399)	7108 (6404‐8339)	10 251 (8613‐12 751)	33 561(30 429‐38 489)

**Table 2:** Total RTI hospital admissions estimated to be due to RSV by the final models, stratified by age group (<6 mo, 6‐11 mo, 1‐4 y) and primary diagnosis, as a percentage of the total hospital admissions for the respective primary diagnosis

Primary diagnosis	Percentage of annual hospital admissions (per primary diagnosis) attributed to RSV (95% CI)			
	<6 mo	6‐11 mo	1‐4 y	Total
Bronchiolitis	82% (79‐87%)	70% (66‐75%)	75% (71‐79%)	78% (75‐83%)
Pneumonia	19% (14‐28%)	22% (19‐25%)	26% (23‐28%)	25% (22‐27%)
Bronchitis	56% (48‐71%)	37% (28‐55%)	14% (8‐20%)	31% (23‐42%)
Unspecified LRTI	6% (3‐8%)	17% (13‐24%)	28% (26‐30%)	24% (22‐27%)
URTI	11% (9‐12%)	8% (6‐12%)	7% (5‐12%)	8% (5‐12%)

**Figure 3:**
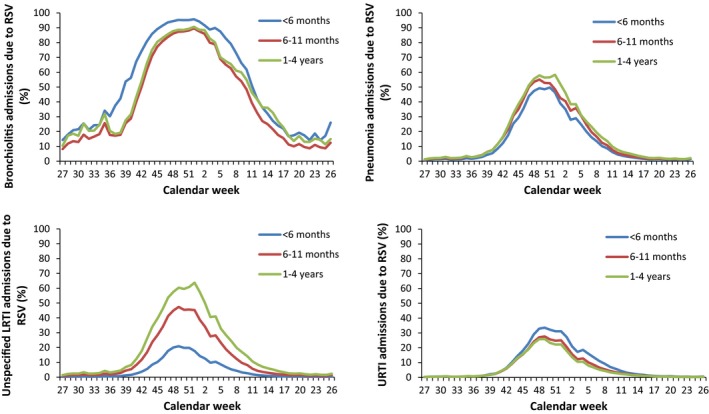
Percentage of total weekly hospital admissions due to RSV for each primary diagnosis, by age group

On average, the estimated admission rate of any RSV‐associated RTI hospital admission was 35.1 (95% CI: 32.9‐38.9) per 1000 children <1 year of age and 5.31 (95% CI: 4.5‐6.6) per 1000 children 1‐4 years of age, per epidemiological year (Table 3). Estimated rates of RSV‐associated hospital admissions were, on average, higher in <1‐year‐olds for all diagnoses except unspecified LRTI, where admission rates in 1‐ to 4‐year‐olds were higher (Table 3). All LRTI admission rates increased in both age groups over time, peaking in 2010/2011 (the RSV season following the 2009 influenza A (H1N1) pandemic, which was an intense seasonal influenza season dominated by A/H1N1pdm09) and with a slight decrease in 2011/2012 for all diagnoses except pneumonia. Admissions for URTI decreased over the study period, particularly in the 1‐4 years of age group which saw a 70% decrease from 2.9 (95% CI: 2.5‐3.4) admissions per 1000 children in 2007/2008 to 0.9 (95% CI: 0.3‐2.1) per 1000 children in 2011/2012. The percentage of weekly hospital admissions attributed to RSV varied by calendar week for all primary diagnoses and age groups (Figure 3).

**Table 3:** Estimated admission rates of RSV‐associated hospital admissions per 1000 children <5 y of age in England

Estimated admission rate of RSV‐associated hospital admissions (per 1000) (95% CI)							
	Age group	2007/8	2008/9	2009/10	2010/11	2011/12	Average
URTI	<1 y	3.00 (2.50‐3.50)	3.41 (2.84‐3.41)	2.73 (2.03‐4.00)	3.00 (2.23‐4.39)	2.59 (1.92‐3.79)	2.95 (2.31‐3.93)
	1‐4 y	2.94 (2.49‐3.38)	3.29 (2.79‐3.29)	0.95 (0.35‐2.27)	1.04 (0.38‐2.48)	0.90 (0.33‐2.14)	1.82 (1.27‐2.81)
Bronchiolitis	<1 y	27.1 (26.2‐28.1)	30.8 (29.8‐31.9)	30.5 (29.2‐33.4)	33.6 (32.1‐36.7)	29.0 (27.7‐31.7)	30.2 (29.0‐32.3)
	1‐4 y	0.64 (0.60‐0.67)	0.72 (0.68‐0.76)	0.87 (0.82‐0.91)	0.94 (0.89‐0.99)	0.82 (0.77‐0.86)	0.90 (0.75‐0.84)
Unspecified LRTI	<1 y	0.40 (0.24‐0.57)	0.46 (0.27‐0.65)	0.93 (0.70‐1.36)	1.03 (0.78‐1.50)	0.89 (0.67‐1.29)	0.74 (0.53‐1.07)
	1‐4 y	1.34 (1.24‐1.45)	1.52 (1.40‐1.64)	1.82 (1.68‐1.97)	1.99 (1.83‐2.15)	1.72 (1.58‐1.86)	1.68 (1.55‐1.81)
Pneumonia	<1 y	0.54 (0.45‐0.63)	0.62 (0.52‐0.72)	0.65 (0.53‐0.84)	0.72 (0.58‐0.93)	2.69 (2.28‐3.16)	1.04 (0.87‐1.26)
	1‐4 y	0.79 (0.71‐0.88)	0.89 (0.81‐0.99)	1.07 (0.96‐1.19)	1.17 (1.05‐1.30)	1.01 (0.91‐1.12)	0.99 (0.88‐1.09)
Bronchitis	<1 y	0.21 (0.18‐0.24)	0.24 (0.20‐0.27)	0.17 (0.13‐0.26)	0.19 (0.15‐0.29)	0.17 (0.13‐0.25)	0.20 (0.16‐0.26)
	1‐4 y	0.02 (0.01‐0.02)	0.02 (0.01‐0.03)	0.02 (0.01‐0.03)	0.02 (0.01‐0.03)	0.02 (0.01‐0.03)	0.02 (0.01‐0.03)
Total	<1 y	31.3 (29.5‐33.0)	35.6 (33.6‐37.6)	35.0 (32.6‐39.8)	38.5 (35.9‐43.8)	35.5 (32.7‐40.1)	35.1 (32.9‐38.9)
	1‐4 y	5.73 (5.05‐6.40)	6.43 (5.67‐7.20)	4.74 (3.82‐6.37)	5.17 (4.17‐6.95)	4.47 (3.61‐6.01)	5.31 (4.46‐6.59)

#### 3.2.2 LRTI admissions: bronchiolitis, pneumonia, bronchitis and unspecified LRTI

Overall, 53% (28 111/53 514, 95% CI: 51‐57%) of LRTI admissions in children <5 years of age could be attributed to RSV, and 54% (15 260/28 111, 95% CI: 52‐58%) of RSV‐associated LRTIs were in infants <6 months old (Table 1). Of RSV‐associated LRTI admissions, 78% (21 830/28 111, 95% CI: 74‐83%) were coded as bronchiolitis, 13% (3766/28 111, 95% CI: 12‐15%) were coded as unspecified LRTI, 8% (2346/28 111, 95% CI: 7‐9%) were coded as pneumonia, and <1% (169/28 111) were coded as bronchitis.

There was an annual average of 27 969 hospital admissions with a primary diagnosis of bronchiolitis in children <5 years of age in England from 2007 to 2012. We estimate that approximately 78% (21 830/27 969, 95% CI: 75‐83%) of these were due to RSV (Tables 1 and 2). There were differences by age, with approximately 82% (14 962/18 246, 95% CI: 79‐87%) of all bronchiolitis admissions in children aged <6 months attributable to RSV, 70% (5319/7652, 95% CI: 66‐75%) of bronchiolitis admissions in children aged 6‐11 months attributable to RSV and 75% (1549/2071, 95% CI: 71‐79%) of bronchiolitis admissions in children aged 1‐4 years attributable to RSV (Table 2). During the period from calendar week 46 to week 2, over 90% of bronchiolitis admissions in children aged <6 months and over 80% in children aged 6‐11 months and 1‐4 years were attributable to RSV each week (Figure 3). The other explanatory pathogens for bronchiolitis admissions were parainfluenza, hMPV and rhinovirus, with differences in causal pathogens by age (see Supplementary Data).

There was an annual average of 9537 hospital admissions with a primary diagnosis of pneumonia in children <5 years of age in England from 2007 to 2012. We estimate that approximately 25% (2346/9537, 95% CI: 22‐27%) of these were due to RSV (Table 1). There were differences by age, with approximately 26% (1923/7503, 95% CI: 23‐28%) of pneumonia admissions in children aged 1‐4 years attributable to RSV compared to 19% in children aged <6 months (138/739, 95% CI: 14‐28%). The other main explanatory pathogens for hospital admissions with a primary diagnosis of pneumonia in children <5 years of age were *S. pneumoniae*, hMPV, parainfluenza, influenza A and rhinovirus, with differences in causal pathogens by age.

There was an annual average of 547 hospital admissions with a primary diagnosis of acute bronchitis in children <5 years of age in England from 2007 to 2012. We estimate that approximately 31% (169/547, 95% CI: 23‐42%) of these were due to RSV (Table 1). There were differences by age, with approximately 56% (89/159, 95% CI: 48‐71%) of bronchitis hospital admissions in children <6 months of age attributed to RSV, compared to only 14% (39/276, 95% CI: 8‐20%) in children aged 1‐4 years (Table 2). The other explanatory pathogens for hospital admissions with a primary diagnosis of bronchitis were parainfluenza and adenovirus in children aged 1‐4 years only.

There was an annual average of 15 461 hospital admissions with a primary diagnosis of unspecified LRTI in children <5 years of age in England from 2007 to 2012. We estimate that approximately 24% (3766/15 461, 95% CI: 22‐27%) of these were due to RSV. There were differences by age, with approximately 28% (3266/11 690, 95% CI: 26‐30%) of unspecified LRTI hospital admissions in children aged 1‐4 years of age attributed to RSV, compared to only approximately 6% (71/1224, 95% CI: 3‐8%) in children <6 months of age (Table 2). The other main explanatory pathogens for hospital admissions with a primary diagnosis of unspecified LRTI in children <5 years of age were adenovirus, *S. pneumoniae*, hMPV, parainfluenza, rhinovirus and influenza A, with differences in causal pathogens by age.

#### 3.2.3 URTI admissions

From 2007 to 2012, there was an annual average of 68 455 hospital admissions with a primary diagnosis of URTI in children <5 years of age in England. We estimate that approximately 8% (5450/68 455, 95% CI: 5‐12%) of these were due to RSV (Table 1). There were no significant differences in the percentage of URTI admissions attributable to RSV by age group, although a slightly higher percentage of admissions in children <6 months of age were attributed to RSV (11%, 942/8868, 95% CI: 9‐12%). The other explanatory pathogens for hospital admissions with a primary diagnosis of URTI were influenza A, parainfluenza and adenovirus.

The number of hospital admissions attributed to each pathogen in each final model is shown in Table S1.

## 4 Discussion

Every year, approximately 33 561 (95% CI: 30 429‐38 489) hospital admissions for RTIs in children <5 years of age from 2007 to 2012 could be attributed to RSV. This represents annual RSV‐associated RTI admission rates of 35.1 (95% CI: 32.9‐38.9) per 1000 children <1 year of age and 5.31 (95% CI: 4.5‐6.6) per 1000 children 1‐4 years of age. The vast majority of these RSV‐associated admissions (84%, 95% CI: 81‐91%) were coded as LRTIs. Of the RSV‐associated LRTIs, nearly half were in children aged <6 months (48%, 95% CI: 46‐52%). Approximately 82% (95% CI: 79‐87%) of hospital admissions for bronchiolitis in children aged <6 months could be attributed to RSV.

Our study is the first to determine burden of RSV‐associated respiratory hospital admissions in children in England according to primary diagnosis and age group. Our study uses smaller age groupings than previous studies to more precisely reflect the differences in RSV‐associated illness by age. Our study is also the first to estimate the burden of RSV in England in the post‐2009 influenza A (H1N1) pandemic era. However, the methods used here do have potential limitations. Firstly, our models assume that the temporal variation in laboratory reports of the causative agents is an accurate representation of their relative incidence over time. The results will be biased if this is not the case (ie due to seasonal changes in laboratory testing or reporting). Secondly, there may be other reasons for temporal variations that are not accounted for in this analysis (eg meteorological variables), and it is possible that some hospital admissions could be attributed to other pathogens with similar temporal patterns to those that we have considered (eg other bacterial pathogens).^10^ Thirdly, it is possible that age‐related differences in testing practices may have impacted our quantitative estimates—particularly those for influenza‐associated admissions. However, that our results demonstrate a significantly higher burden of RSV‐associated hospital admissions in young children compared to other respiratory pathogens, including influenza, is consistent with the results of previous studies.^16^, ^17^, ^18^ Finally, as we restricted hospital admissions to those with a primary diagnosis of URTI or LRTI, our results are likely to be an underestimate of the true burden of RSV‐associated hospital admissions.

Previous estimates of RSV burden in secondary care in England range from 26 500 to 29 160 RSV‐associated hospital admissions per year in children <5 years of age—all lower than our estimate of 33 561 (95% CI: 30 429‐38 489) RSV‐associated admissions, which is probably due to those studies considering earlier time periods.^4^, ^5^, ^6^, ^10^ Our study found a steady, general increase in the admission rates of RSV‐associated LRTI hospital admissions from 2007/2008 to 2011/2012, peaking during the 2010/2011 season. There are a number of potential explanations for this increase over time. Our models are sensitive to the number of positive laboratory tests; therefore, our results could be affected by a change in the relative sensitivity or specificity of the assays for the different pathogens over time, although the majority of laboratories have been using RT‐PCR for most of the study period. However, from 1999 to 2010, there has been an increase of 28% in emergency hospital admissions in children <5 years of age, particularly admissions for acute illness, and the annual number of hospital admissions due to bronchiolitis in young children in the UK has also increased sevenfold between 1979 and 2011.^8^, ^19^ This evidence suggests that the increase is not due to an increase in the severity of infection, nor the virulence of RSV in the age group, because paediatric intensive care admission rates have changed little from 2004 to 2012.^8^ Instead, these trends are likely due to a general increase in hospital admission, potentially due to hospital admission thresholds being lowered (particularly in the younger infants) or failure to manage these acute illnesses in the community care setting.^20^

Our study found a high burden of RSV‐associated hospital admissions in children <6 months of age, a group well documented as being at high risk of RSV‐associated hospital admission**.** 21 However, our study also found a high number of RSV‐associated pneumonia and unspecified LRTI admissions in children aged 1‐4 years. RSV was the pathogen associated with the highest number of admissions for all types of LRTI in all age groups compared to the other pathogens, except for pneumonia and unspecified LRTI in children aged <6 months and 6‐11 months. It is therefore possible that RSV‐associated hospital admissions are more likely to be coded as bronchiolitis in young infants, but as pneumonia or unspecified LRTI in children older than one year.

As this analysis is at the population level, it is not possible to investigate individual‐level risk factors for RSV‐associated hospital admission or additional outcomes indicating the severity of illness such as length of stay or admission to paediatric intensive care. Individual‐level analysis of RSV‐associated hospital admission could be achieved through linkage of laboratory surveillance data and hospital admissions data, as demonstrated in Western Australia.^22^

In conclusion, RSV is a significant cause of hospital admissions for LRTI in children <5 years of age in England. Large‐scale individual‐level analysis of RSV‐associated hospital admissions in young children in secondary care in England is required to compare the severity of cases by age. This work provides a baseline for vaccine impact studies if such vaccines are introduced into the UK immunisation programme in the future.
