# Supplementary material for: Estimating the burden of respiratory syncytial virus (RSV) on respiratory hospital admissions in children less than five years of age in England, 2007‐2012
Source: Influenza Other Respir Viruses. 2017 Jan 21;11(2):122–9. doi: 10.1111/irv.12443 (PMC5304572; doi:10.1111/irv.12443)
Supplement: Supplementary file 1 [file IRV-11-122-s001.docx]

Supplementary Data

This document provides supplementary data to “Estimating the burden of respiratory syncytial virus (RSV) on respiratory hospital admissions in children less than five years of age in England, 2007-2012” by RM Reeves et al.

Supplementary Table 1 shows the explanatory pathogens included in each final model, and details the average annual estimated hospital admissions in children <5 years of age in England attributed to each pathogen (RSV, influenza A, influenza B, rhinovirus, parainfluenza, hMPV, Adenovirus, *S. pneumoniae*, *M. pneumoniae*, *H. influenza*) by the final models, stratified by age group (<6 months, 6-11 months, 1-4 years).

Supplementary Figures 1 to 3 illustrate the fit of the models for (1) bronchiolitis, (2) pneumonia, and (3) unspecified lower respiratory tract infection (LRTI), by showing the weekly number of hospital admissions in England from 2007-2012 due to each pathogen, as estimated by the final models, in: (a) children aged <6 months; (b) children aged 6-11 months; (c) children aged 1-4 years.

Supplementary Table S1. Average annual estimated hospital admissions in children <5 years of age in England attributed to each pathogen (RSV, influenza A, influenza B, rhinovirus, parainfluenza, hMPV, Adenovirus, *S. pneumoniae*, *M. pneumoniae*, *H. influenza*) by the final models, stratified by age group (<6 months, 6-11 months, 1-4 years).

| **Average annual number of hospital admissions**  **(95% CI)** | | | | | | | | | | | | | | | |
| --- | --- | --- | --- | --- | --- | --- | --- | --- | --- | --- | --- | --- | --- | --- | --- |
|  | **Bronchiolitis** | | | **Pneumonia** | | | **Bronchitis** | | | **Unspecified LRTI** | | | **URTI** | | |
|  | **<6m** | **6-11m** | **1-4y** | **<6m** | **6-11m** | **1-4y** | **<6m** | **6-11m** | **1-4y** | **<6m** | **6-11m** | **1-4y** | **<6m** | **6-11m** | **1-4y** |
| Total | 18246 | 7652 | 2071 | 739 | 1294 | 7503 | 159 | 113 | 276 | 1225 | 2546 | 11690 | 8868 | 12737 | 26280 |
| **Explained by:** | | | | | | | | | | | | | | | |
| RSV | 14962 (14396 - 15942) | 5319 (5066 - 5775) | 1549 (1467 - 1631) | 138 (101 - 204) | 285 (245 - 324) | 1923 (1718 - 2128) | 89 (76 - 113) | 41 (29 - 62) | 39 (23 - 55) | 71 (37 - 104) | 429 (322 - 619) | 3266 (3007 - 3525) | 942 (802 - 1082) | 1034 (742 - 1559) | 3474 (2398 - 5412) |
| Influenza A | - | - | - | - | 54 (9 - 168) | 115 (23 - 207) | - | - | - | 28 (12 - 45) | 51 (43 - 127) | - | 323 (74 - 774) | 362 (272 - 453) | 1259 (886 - 1633) |
| Influenza B | - | - | - | - | - | - | - | - | - | - | - | - | - | - | - |
| Rhinovirus | - | 1640 (1185 - 2096) | 279 (79 - 479) | - | - | 1914 (529 - 4157) | - | - | - | - | 251 (174 - 966) | 2731 (708 - 5982) | - | - | - |
| Parainfluenza | 1598 (902 - 2293) | 505 (199 - 811) | - | 112 (66 - 158) | 236 (167 - 304) | 736 (416 - 1056) | - | - | 33 (1 - 64) | 214 (121 - 369) | 440 (350 - 530) | 1040 (560 - 1521) | 1046 (623 - 1742) | 917 (594 - 1240) | 2612 (1281 - 3944) |
| hMPV | 593 (204 - 982) | - | - | 56 (30 - 81) | 105 (70 - 143) | 469 (279 - 659) | - | - | - | 114 (79 - 148) | 323 (269 - 377) | 612 (318 - 907) | - | - | - |
| Adenovirus | - | - | - | - | - | - | - | - | 108 (38 - 179) | - | - | 1911 (272 - 3551) | - | 3408 (2159 - 5201) | 15187 (11881 - 18494) |
| *S. pneumoniae* | - | - | - | 349 (140 - 558) | 550 (240 - 860) | - | - | - | - | 566 (300 - 832) | 432 (35 - 829) | - | - | - | - |
| *M. pneumoniae* | - | - | - | - | - | - | - | - | - | - | - | - | - | - | - |
| *H. influenza* | - | - | - | - | - | - | - | - | - | - | - | - | - | - | - |

“ - ” denotes that the pathogen was not included in the final model.

**a**

**b**

**c**

Supplementary Figure 1. Weekly bronchiolitis hospital admissions due to respiratory syncytial virus (RSV), parainfluenza and human metapneumovirus (hMPV) in England from 2007-2012, as estimated by the final models, in: (a) children aged <6 months; (b) children aged 6-11 months; (c) children aged 1-4 years. †

**a**

**b**

**c**

Supplementary Figure 2. Weekly pneumonia hospital admissions due to respiratory syncytial virus (RSV), parainfluenza, human metapneumovirus (hMPV), *S. pneumonia*, influenza A and rhinovirus in England from 2007-2012, as estimated by the final models, in: (a) children aged <6 months; (b) children aged 6-11 months; (c) children aged 1-4 years. †

**a**

**b**

**c**

Supplementary Figure 3. Weekly unspecified LRTI hospital admissions due to respiratory syncytial virus (RSV), influenza A, parainfluenza, human metapneumovirus (hMPV), *S. pneumonia*, rhinovirus and adenovirus in England from 2007-2012, as estimated by the final models, in: (a) children aged <6months; (b) children aged 6-11 months; (c) children aged 1-4 years. †

*† Only viruses significantly contributing to the age-group specific model (and therefore included in the final model) are included in the figures.*
